# Neighborhood Variation in Rate of Revascularization among Acute Myocardial Infarction Patients in New York City

**DOI:** 10.4061/2011/348527

**Published:** 2011-10-19

**Authors:** Abdissa Negassa, Jing Fang

**Affiliations:** ^1^Division of Biostatistics, Department of Epidemiology and Population Health, Albert Einstein College of Medicine, Bronx, NY 10461, USA; ^2^Division of Epidemiology, Department of Epidemiology and Population Health, Albert Einstein College of Medicine, Bronx, NY 10461, USA

## Abstract

*Objective*. To identify modifiable neighborhood factors and quantify their effect on the rate of revascularization among acute myocardial infarction (AMI) patients. *Method*. Using the New York City hospital discharge records during 1998–2002, we employed a hierarchical regression model that integrates patient-level risk factors and neighborhood-level factors to retrospectively examine revascularization patterns among AMI patients. *Results*. Access to revascularization varied substantially (27%–88%) among neighborhoods. Ready access to a hospital with on-site capacity of revascularization increased the likelihood of receiving the procedure after adjusting for individual-level sociodemographic factors and comorbidity. More than 64% of the variation in rate of revascularization is explained by access to revascularization. *Conclusion*. Optimizing the AMI patients' delivery system to hospitals with on-site capacity of revascularization might enhance access to needed care thereby help to alleviate the prevailing variation in the rate of revascularization among New York City neighborhoods.

## 1. Introduction

Invasive cardiac revascularization procedures, including percutaneous transluminal coronary angioplasty (PTCA) and coronary artery bypass graft surgery (CABG), can improve the outcome of coronary heart disease [1–4]. In a metropolitan urban setting, residency may influence utilization. Inequitable geographic distributions of health care professionals, hospitals, and services provided by these hospitals exist throughout the USA [5]. In New York City (NYC), for example, only few hospitals are licensed to perform cardiac revascularization and they are not uniformly distributed. It has been reported that in some neighborhoods in NYC, due to the unavailability of hospitals performing revascularization in the neighborhood, patients with acute myocardial infarction (AMI) were less likely to undergo revascularization than those living in neighborhoods with hospitals performing revascularization [6]. Nonetheless, whether this is generally the case throughout NYC and what aspect of residency impacts the odds of getting revascularization the most, above and beyond key patient characteristics, is unknown.

In this paper, we report on how aspects of residency influence revascularization among AMI patients in NYC by integrating patient-level as well as neighborhood-level data.

## 2. Methods

### 2.1. Data Sources

We used the 1998–2002 Statewide Planning and Research Cooperative System (SPARCS) database, created and maintained by the New York State Department of Health [7]. The SPARCS database is legislatively mandated and contains discharge data abstracted for at least 95% of all New York State hospital admissions except from psychiatric and federal hospitals. The SPARCS database includes data on age, sex, race, zip code of residence, admission status, physician and hospital identifiers, principal diagnosis and up to 14 secondary diagnoses, principal procedure code and up to 14 other procedure codes, and discharge status. Diagnoses and procedures are coded by the International Classification of Disease, 9th Revision, Clinical Modification (ICD-9-CM). Trained personnel abstract data from medical records in each hospital and the New York State Department of Health verifies the accuracy of reported information.

AMI was defined by principal diagnosis with ICD-9-CM codes 410.0 through 410.9. Since AMI rarely occurs before age 35, this study was restricted to patients with age ≥35 years and is based on 72,188 records of AMI patients. Revascularization was defined by any procedure codes, including PTCA (ICD 36.01, 36.02, 36.05, 36.06) and CABG (ICD 36.10 through 36.19). Cardiovascular comorbidity was defined by ICD-9 codes from secondary diagnoses, and included diabetes (ICD 250), congestive heart failure (CHF) (ICD 428.0, 428.2–428.4), hypertension (ICD 401–405), hyperlipidemia (ICD 272.0–272.4), obesity (ICD 278.0), renal disease (ICD 585.1–585.5), and pulmonary disease (ICD 490–496).

### 2.2. Patient-Level Data

We considered patient's age, gender, ethnicity, insurance status, and comorbidities such as hypertension, diabetes, and congestive heart failure (CHF), in the analysis of variation in rate of revascularization. Ethnicity was defined based on Hispanic origin. Therefore, in our subsequent analysis, we recoded ethnicity as dichotomous, that is, non-Hispanic white versus others. Insurance status was based on primary and secondary coverage and was categorized as (i) private insurance (alone or with public insurance); (ii) public insurance (Medicare, Medicaid, and other government insurance); (iii) no insurance. Previous studies have identified these variables as strong predictors of the probability of undergoing cardiac revascularization [8–11].

### 2.3. Neighborhood-Level Data

We grouped patients by spatially defined neighborhoods. Neighborhoods were created by combining adjoining zip codes sharing similar socioeconomic characteristics as per the United Hospital Fund [12]. This resulted into 41 neighborhoods in NYC: 4 in Staten Island, 7 in the Bronx, and 10 each in Brooklyn, Manhattan, and Queens. This neighborhood delineation is used by the NYC Department of Health for community health surveys and reflects catchment areas for certain healthcare facilities. This definition of neighborhood is appropriate for assessing access to health care [13]. 

We considered neighborhood variables such as the percentage of patients with CHF, diabetes, and health insurance. These variables are employed to assess neighborhood composition and as a proxy for the social, economical and physical/environmental aspects of the respective neighborhoods. For instance, the percentage of patients with obesity or diabetes might reflect the extent of access to fresh food or quality municipal services such as parks conducive to healthy physical activity or the prevailing attitude within a neighborhood towards healthy living in general. In addition, for each neighborhood we defined a new variable measuring access to coronary revascularization for its residents. Access to coronary revascularization is computed as the percentage of AMI patients who had their *index admission* for treatment of myocardial infarction at a hospital with on-site capacity of revascularization. This definition of access was also adopted by others [11, 14]. In order to determine hospital location, the SPARCS database hospital code was linked with hospital name. Neighborhood level income data was derived from the US Census data (2000) based on zip code.

### 2.4. Statistical Analysis

We are interested in examining how the odds of revascularization is influenced not only by patient's presenting characteristics such as age, gender, insurance, and comorbidity but also how it varies by neighborhood and the role of neighborhood characteristics in this variation. Therefore, we need to take into account two sources of variation: variation due to differences in patients within a neighborhood and variation due to between neighborhood differences. 

We employed a two-level hierarchical logistic regression model. Modeling *p*_*ij*_ the probability of undergoing revascularization for the *j*th patient in the *i*th neighborhood with

(1) $$\begin{array}{c} \text{logit}\left(p_{ij}\right)=b_{0i}+b_{1}x_{1}+b_{2}x_{2}+\cdots +b_{k}x_{k},\\ b_{0i}=b_{0}+\upsilon_{i},\\ \upsilon_{i}\sim N\left(0,\sigma^{2}\right), \end{array}$$

where the *x*'s represent the patient's age, gender, ethnicity, insurance status, and comorbidities and *b_0i_* is the log of the background odds of revascularization which is allowed to vary from neighborhood to neighborhood. The variability in *υ*_*i*_ represents the variability that can be attributed to neighborhood-level factors after taking into account patient-level covariates that are included in the model. A crude estimate of rate of revascularization is obtained by dividing the number of patients with AMI who underwent the procedure in a given neighborhood by the total number of patients who suffered AMI in the same neighborhood over the study period.

Subsequently, we extended the above model by sequentially including neighborhood-level factors. This allowed us to assess the extent of variation in *υ*_*i*_ attributable to a given neighborhood-level factor. In the following, we refer to the variation in *υ*_*i*_ as neighborhood variation. The assessment was done by computing the relative reduction in neighborhood variation when a given neighborhood-level factor was included in the model.

While interpretation of the effect of individual-level factors/covariates is relatively simple, that is, the usual odds ratio interpretations apply for comparison of individuals belonging to the same neighborhood, the interpretation of the effect of neighborhood-level factors/covariates is not immediately obvious. The difficulty arises from the need to compare individuals from different neighborhoods. Two quantities, median odds ratio (MOR) and interval odds ratio (IOR) have been proposed in the literature in order to address this problem [15]. These quantities facilitate the interpretation of neighborhood variation in terms of the familiar odds ratio.

In our case, the MOR quantifies variation between neighborhoods by comparing two randomly chosen individuals from two neighborhoods. It is the median odds ratio between the individual from a neighborhood with high access to revascularization and another individual from a neighborhood with low access to revascularization. Large MOR signifies heterogeneity. Likewise, the IOR is proposed as a fixed-effects measure to further quantify the effect of neighborhood-level covariates [15]. 

## 3. Results

In this study of patients with AMI admitted to NYC hospitals between 1998–2002, the crude rate of revascularization ranged between 20%–47% among NYC neighborhoods, with a median of 29.7%. There was large variation among the neighborhoods with respect to number of residents, ranged between 29,266–464,736, and per capita income, ranged between $8,732–$70,625, according to US Census data (2000). There was also moderate variation in patient characteristics and comorbidity across the neighborhoods (see Table 1). 

**Figure 1:**
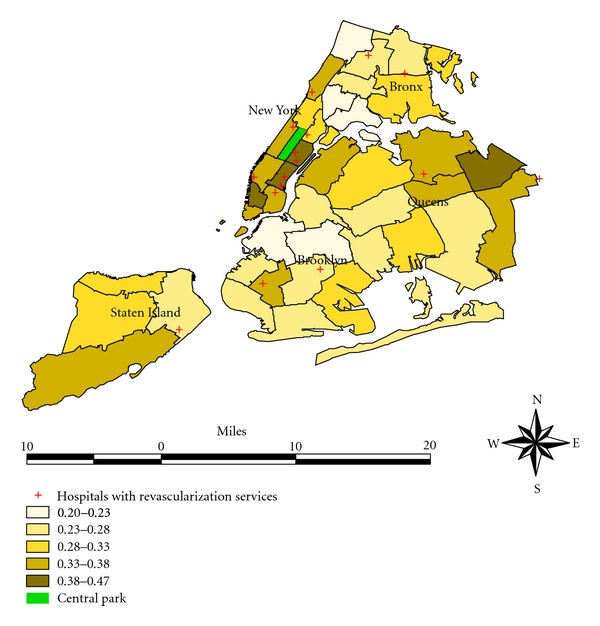
Crude rate of revascularization after AMI in NYC neighborhoods1998–2002.

**Figure 2:**
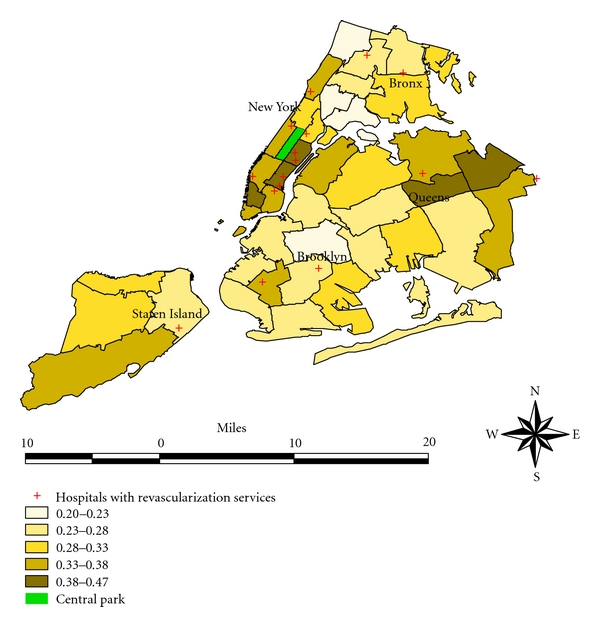
Adjusted rate of revascularization after AMI in NYC neighborhoods 1998–2002.

Access to revascularization also exhibited considerable variation across neighborhoods. There were only 16 hospitals with on-site revascularization capacity located within 14 neighborhoods (Figure 1). The presence of a hospital with an on-site capacity of revascularization within a neighborhood did not necessarily lead to a higher observed rate of revascularization in the corresponding neighborhood (Figure 1). Even after adjusting for patient's characteristics, the presence of a hospital with on-site capacity of revascularization within a neighborhood still did not necessarily correspond to a higher rate of revascularization (Figure 2). On the other hand, living in a neighborhood with a high rate of index admission to a hospital with on-site capacity of revascularization substantially increased the odds of undergoing revascularization after adjusting for key patient characteristics that are known to be related to revascularization (Tables 2 and 3). 

In our analysis, what is important is not just the mere statistical significance of the effect of a given neighborhood-level covariate but its contribution in light of the variability associated with the background odds of revascularization. Therefore, we sequentially added the neighborhood-level covariates into the model one at a time and assessed the subsequent relative change in neighborhood variation. As shown in Table 3, the largest relative reduction in neighborhood variation was achieved when the neighborhood-level covariate access to revascularization was included in the model, that is, 64.31%. This was followed by the percentage of patients with diabetes, resulting in a relative reduction of 46.37% in neighborhood variation and then by poverty level as assessed by percentage of households below poverty line, with a relative reduction of 42.71%. After adjusting for patient-level covariates, the MOR was 1.49, indicating the presence of appreciable heterogeneity.

**Figure 3:**
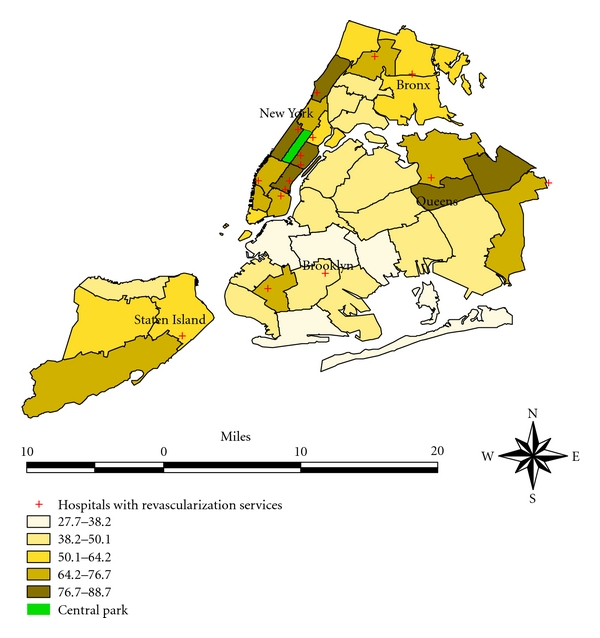
Pattern of access to revascularization after AMI in NYC neighborhoods 1998–2002.

In order to further characterize this heterogeneity, we computed IOR. In Table 3, we considered two hypothetical individuals from two different neighborhoods that differ substantially in access to revascularization, say falling in the 1st and 4th quartiles, respectively. To put it in context, this translates into at least 30 percentage points difference in access to revascularization; this neighborhood-level covariate ranged from 27.7% to 88.7%. From Table 3, the IOR associated with access to revascularization was (1.51, 3.72). This interval also indicates that access to revascularization accounted for an appreciable portion of neighborhood variation. An inverse association was suggested with respect to the percentage of patients with diabetes (IOR: 0.26, 0.80) and percentage of households below poverty line (IOR: 0.26, 0.83). These neighborhood-level covariates also explained sizable portion of neighborhood variation, next to access to revascularization (see Table 3). In contrast, a similar computation revealed that the percentage of patients with CHF is not that important in light of the high residual neighborhood variation. Similar observation was made for the rest of the neighborhood-level covariates considered in this study (see Table 3). Considering all available neighborhood-level covariates simultaneously, by employing the model that resulted in the lowest residual neighborhood variation, did not explain away the effect of access to revascularization, (IOR: 1.31, 2.63), while reducing the effects of percent diabetes and poverty level to borderline, (IOR: 0.53, 1.07) and (IOR: 0.49, 1.00), respectively. The MOR also concurred with IOR in that when access to revascularization was included in the model, the residual heterogeneity reduced substantially (see Table 3). The pattern of access to revascularization, that is, percentage of AMI patients with index admission to a hospital with on-site revascularization capacity, by neighborhood, in NYC is shown in Figure 3.

## 4. Discussion

Our analysis of revascularization among AMI patients in NYC between 1998–2002 by taking into account both patient-level and neighborhood-level covariates revealed that access to revascularization, as assessed by the percentage of index admission to a hospital with on-site capacity, substantially increases the odds of subsequently receiving revascularization. To our knowledge, this is the first analysis integrating patient-level and neighborhood-level covariates in assessing variability in procedure utilization among AMI patients in a large metropolitan setting such as New York City.

Regarding patient's characteristics, our result is consistent with previous findings in that age, gender, ethnicity, and insurance were strong predictors of the likelihood of undergoing revascularization. In addition, almost all of the comorbidities we considered, except hyperlipidemia and hypertension, were associated with lower odds of undergoing revascularization. This might be a reflection of some sort of selection in terms of providing the procedure on the basis of comorbidity profile or patients with these comorbidities might be sicker and less suitable candidates for revascularization. 

Our finding suggests that modifying factors impacting index admission to a hospital with on-site capacity might enhance the odds of subsequently getting revascularization. One such potential factor is transportation of an AMI patient to a hospital with on-site capacity. Previously it was reported that, although practices vary in New York State, emergency medical transport rules often direct that patients with acute chest pain be taken to the nearest hospital [8]. However, when hospitals with on-site capacity, especially greater-volume, are located within 30 minutes distance, instructing emergency transport teams to take an AMI patient to the nearest such hospital would enhance the patient's chance of receiving revascularization and subsequent better clinical outcome [16]. On the other hand, there might be settings in which a strategy involving initial stabilization of an AMI patient in a facility without revascularization capabilities and subsequent transfer to a hospital with revascularization capabilities would be more appropriate.

Our observation also substantiates the findings of previous studies that reported the shorter the distance to a hospital with on-site capacity of revascularization, the higher the likelihood of index admission to such a hospital and subsequently getting revascularization [8, 17, 18]. Having such hospitals in every neighborhood might be impractical; however, devising an efficient system of delivering AMI patients to the appropriate hospital is more feasible. The current policy on prehospital transportation of an AMI patient in New York City is to transport the patient to the closest 24 hours New York State certified interventional cardiac catheterization facility unless the patient has other medical conditions that warrant transportation to the closest hospital emergency department [19]. 

The use of an administrative database is a potential limitation of this study. Errors in diagnostic and procedure coding could possibly have impacted our results. However, given the rigorous quality assurance procedures of the SPARCS database [7], we believe that in this standardized database, such errors are relatively small. Moreover, under the more plausible assumption of nondifferential misclassification, our result would be conservative.

Hospital affiliation with other hospitals, payer groups, and primary care physician affiliation play a role in influencing procedure utilization [18]. In addition, time wasted in transferring an AMI patient to another hospital with on-site capacity of revascularization might be an additional deterring factor but the time delay of interhospital transportation seems to be not an important factor with respect to clinical outcome, once the patient received revascularization [20]. Our observation of a relatively lower revascularization rate in one of the neighborhoods with revascularization facility, Staten Island with a new revascularization facility in 2000, might be the result of long-established referral patterns remaining strong determinants of revascularization use even though resources became available within the neighborhood [14].

In conclusion, our findings suggest that having ready access to a hospital with on-site capacity of revascularization increases the odds of subsequently receiving the procedure. This result is adjusted for patient-level covariates known to be associated with the likelihood of undergoing revascularization and other neighborhood-level covariates. Therefore, optimizing the efficiency of AMI patients' delivery system to a hospital with on-site capacity might potentially enhance receiving the needed care; thereby help to alleviate the prevailing variation in the rate of revascularization among New York City neighborhoods.

## Figures and Tables

**Table 1:** Descriptive summary of patient characteristics and comorbidity in NYC neighborhoods, 1998–2002.

Characteristics	Median	Minimum	Maximum
Average age	69.0	64.0	73.0
Male*	45.2%	40.8%	53.2%
Nonwhite	59.0%	12.0%	99.0%
CHF	33.0%	24.0%	45.0%
Obesity	1.5%	0.4%	2.6%
Diabetes	34.0%	19.0%	47.0%
Hypertension	58.0%	50.0%	70.0%
Renal disease	3.1%	0.6%	4.9%
Hyperlipidemia	23.0%	15.0%	30.0%
Pulmonary disease	13.0%	9.0%	20.0%
Public insurance	46.0%	23.0%	77.0%
Poverty level**^†^**	18.4%	5.2%	45.6%
Access to revascularization	57.0%	27.7%	88.7%

*The denominator is the total number of patients with AMI within a neighborhood.

**^†^**Percentage of households below poverty line.

**Table 2:** Odds ratio (OR) and 95% confidence interval (CI) for patient-level covariates.

Patient-level covariates	OR	95% CI
Age		
35–40	1.00	—
40–50	1.21	(1.05,1.39)
50–60	1.19	(1.04, 1.37)
60–70	1.08	(0.94, 1.23)
70–80	0.88	(0.77, 1.00)
80+	0.35	(0.30, 0.40)
Male	1.39	(1.34,1.44)
Nonwhite	0.74	(0.71, 0.77)
CHF	0.63	(0.60, 0.65)
Obesity	0.90	(0.78, 1.04)
Diabetes	0.91	(0.88, 0.94)
Hypertension	1.17	(1.13, 1.22)
Renal disease	0.60	(0.54, 0.68)
Hyperlipidemia	1.92	(1.85, 2.0)
Pulmonary disease	0.78	(0.74, 0.83)
Insurance		
Private	1.96	(1.81, 2.13)
Public	1.59	(1.48, 1.73)

**Table 3:** Assessment of neighborhood-level covariates (adjusted for selected patient-level covariates as shown in Table 2).

Neighborhood-level covariates	MOR*	Δ in MOR (%)^†^	Δ in Variance (%)^‡^	IOR
CHF	1.49	−0.30	−1.50	(0.56, 2.56)
Obesity	1.42	4.08	19.93	(0.29, 1.14)
Diabetes	1.34	9.87	45.59	(0.26, 0.80)
Hypertension	1.41	4.82	23.41	(0.31, 1.17)
Renal disease	1.43	3.69	18.08	(0.32, 1.26)
Hyperlipidemia	1.49	−0.63	−3.19	(0.42, 1.96)
Pulmonary disease	1.50	−0.92	−4.69	(0.44, 2.07)
Public insurance	1.40	6.06	29.08	(0.29, 1.04)
Poverty level	1.35	9.18	42.71	(0.26, 0.83)
Access to Revascularization	1.27	14.75	64.31	(1.51, 3.72)

Abbreviation: IOR: interval odds ratio; MOR: median odds ratio.

*The MOR derived from the model containing patient-level covariates only is 1.49.

**^†^**Change in MOR relative to the one derived from the model containing patient-level covariates only.

^‡^Change in neighborhood variation relative to the model containing patient-level covariates only.
